# Development and Validation of a Mobile-Centered Digital Health Readiness Scale (mDiHERS): Health Literacy and Equity Scale

**DOI:** 10.2196/58497

**Published:** 2024-08-13

**Authors:** Hana Kim, Rebecca Schnall, Nagyeom Yoon, Seong-Joon Koh, Jisan Lee, Jae Hee Cheon

**Affiliations:** 1 Department of Nursing Hoseo University Asan Republic of Korea; 2 School of Nursing Columbia University New York, NY United States; 3 Department of Nursing Gangneung-Wonju National University Wonju Republic of Korea; 4 Department of Internal Medicine and Liver Research Institute Seoul National University College of Medicine Seoul Republic of Korea; 5 Department of Internal Medicine Yonsei University College of Medicine Seoul Republic of Korea

**Keywords:** digital health, health literacy, health equity, inflammatory bowel diseases, telemedicine, patient participation, validation, validate, IBD, bowel, inflammatory, inflammation, gastrointestinal, GI, internal medicine, gastroenterology, scale, readiness, adoption, measure, measures, measurement, measurements, assessment, assessments, scales, eHealth, e-health, literacy, mHealth, mobile health, chronic, mobile phone

## Abstract

**Background:**

There has been a rapid expansion of digital health care services, making the need for measuring and improving digital health readiness a priority. In response, our study team developed the *Mobile-Centered Digital Health Readiness: Health Literacy and Equity Scale* (mDiHERS) to measure digital health readiness.

**Objective:**

We aim to develop and validate a scale that assesses digital health readiness, encompassing literacy and equity, and to ensure the effective use of mobile-centered digital health services.

**Methods:**

This study was conducted from October 2021 to October 2022 to develop and validate the mDiHERS. Participants included patients with inflammatory bowel disease, which is a chronic condition requiring continuous management, and experts in medical and nursing informatics. The scale development involved a literature review, focus group interviews, and content validity evaluations. A total of 440 patients with inflammatory bowel disease were recruited for the validation phase, with 403 completing the survey. The scale’s validity and reliability were assessed through exploratory factor analysis and Cronbach α. The scale was translated into English by translators and bilingual and native researchers, ensuring its applicability in diverse settings.

**Results:**

The mDiHERS consists of 36 items across 6 domains, with a 5-point Likert scale for responses. The validation process confirmed the scale’s construct validity, with 4 factors explaining 65.05% of the total variance. The scale’s reliability was established with Cronbach α values ranging from 0.84 to 0.91. The scale’s development considered the technical proficiency necessary for engaging with health mobile apps and devices, reflecting the importance of subjective confidence and objective skills in digital health literacy.

**Conclusions:**

The mDiHERS is a validated tool for measuring patients’ readiness and ability to use digital health services. The mDiHERS assesses user characteristics, digital accessibility, literacy, and equity to contribute to the effective use of digital health services and improve accessibility. The development and validation of the mDiHERS emphasize the importance of confidence and competence in managing health digitally. Continuous improvements are necessary to ensure that all patients can benefit from digital health care.

## Introduction

The digital health care service market has experienced significant expansion. Digital health, as defined by the US Food and Drug Administration, represents the convergence of people, information, technology, and connectivity, all of which collaboratively enhance health care delivery and outcomes. This extensive field includes various components such as mobile health (mHealth), health information technology, wearable devices, telehealth and telemedicine, and personalized medicine, each playing a crucial role in advancing the functionality and reach of contemporary medical practices [1].

The evolution of digital health is further propelled by patients who, as informed health consumers, are increasingly harnessing digital devices for health management [2]. Digital health services, as defined in this study, refer to the use of digital health to manage and monitor patient health outcomes, complementing the accessible, efficient, and patient-centric delivery of digital health [3]. A notable advancement in this domain is remote health managing and monitoring, which empowers patients to access health care services within the comfort of their homes, thereby fostering a sense of independence [3,4].

Increased use of digital health services has the potential to induce disparities in accessibility, proficiency, and the degree of health information and technology use among user groups, necessitating careful consideration of vulnerable populations. In response to these challenges, there is a growing emphasis on devising strategies to gauge the digital divide and implement system-wide solutions that champion digital inclusion, ensuring that marginalized groups are not left behind in the digital health landscape [5].

However, to support equitable provision of digital health services, prioritization should be given to measuring the digital divide rather than merely focusing on the means of delivery [6]. “Digital readiness” encompasses digital access, use, literacy, and the competency to engage with digital health services [7]. It emerges as a pertinent metric to assess disparities in digital health service use [7].

Existing digital health literacy scales have primarily focused on specific aspects. For instance, the Digital Health Literacy Instrument evaluates internet usage skills related to health on the web and computers but does not consider aspects related to mobile usage [6]. Similarly, the eHealth Literacy Scale assesses the ability to find, evaluate, and apply electronic health information to health issues but does not evaluate the importance of mHealth apps and devices and the ability to use mobile services required for daily life [8]. These limitations may result in an inaccurate reflection of users’ digital health readiness. Additionally, because existing health literacy scales rely on subjective measurements, it is impossible to know how an individual’s responses relate to actual skill level, whereas objective tests can directly measure an individual’s skills [9,10]. Motivated by this, our study endeavors to construct and validate a scale measuring digital health readiness consisting of subjective and objective questions.

One of the core principles of Healthy People 2020 is to eliminate health disparities and achieve health equity to attain health and well-being [11]. As the health care sector becomes digitized, digital access, such as through mobile apps, is now recognized as a social determinant of health [12]. Low digital access due to low digital literacy undermines health equity [13]. Despite the development and research of many tools to improve and measure equity, most of these tools only measure equity in health care settings and public health domains [14-16]. Specifically, the Health Equity Assessment Toolkit was developed for use primarily by public health professionals, researchers, and those with basic skills in health information systems and interpreting health-related data, rather than by the target population directly [17]. Therefore, our study developed measures to assess health equity based on digital literacy.

Inflammatory bowel disease (IBD), encompassing Crohn disease and ulcerative colitis, is characterized by chronic digestive tract inflammation. This condition leads to a range of symptoms that significantly impact the quality of life [18]. The persistent symptoms and activity of IBD, even with the best medical or surgical interventions, highlight the critical need for ongoing surveillance and management [19]. Given the complex, chronic nature of IBD, which significantly impairs quality of life, there have been various efforts to leverage technologies to modify behaviors and assist in the self-management of patients with IBD [20,21]. As of 2023, while over 40 free English-language IBD mobile apps have been found to meet acceptable quality criteria, there is a crucial need for enhanced design features to improve user interest and engagement [22]. Recognizing the importance of continuous and personalized interventions, which focus on the immediate management of IBD symptoms [23], it becomes essential to evaluate patients’ access to and understanding of mobile technology about the available apps. This study, therefore, seeks to create an instrument for evaluating digital health preparedness, with an initial focus on individuals with IBD. This demographic is not only in dire need of digital health solutions but also exhibits a higher demand for such services. While first tested for specific conditions, the assessment tool is designed to be flexible and applicable across a spectrum of health scenarios [24]. Consequently, this instrument could be applied to a broad range of patient groups beyond IBD in subsequent studies, aiming to bridge the digital health divide.

## Methods

### Study Design

#### Overview of Study Design

This study was conducted from October 8, 2021, to October 7, 2022. This study was developed through a 4-step process. Step 1 involved a literature review to derive the initial scale items related to digital health literacy and digital health equity. Step 2 aimed to gain qualitative insights into the initial scale items by conducting focus group interviews (FGIs) with 6 patients with IBD and 6 experts, followed by an evaluation of the content validity of the initial scale items by 8 experts. Step 3 evaluated the validity and reliability of the final scale items quantitatively with 440 patients with IBD. This included survey research and statistical analysis. Step 4 translated the final scale items into both English and Korean simultaneously.

#### Participants and Inclusion Criteria

In all stages, patient participants were required to meet the following criteria: (1) have a confirmed diagnosis of ulcerative colitis or Crohn disease, (2) possess the ability to use a smartphone proficiently, (3) be adults aged 18 years or older, and (4) be willing and able to provide informed consent. Additionally, participants needed to be capable of completing FGIs or surveys as required in each stage.

#### Recruitment

Participant recruitment was conducted from November 30, 2021, to September 30, 2022. Patients with IBD were recruited in steps 2-1, 2-3, and 3-1. Recruitment was conducted in the outpatient clinic of the gastroenterology department at S Hospital in Seoul and through the IBD online community in the social network service (eg, KakaoTalk; Kakao Corp). Posters introducing this study, approved by the institutional review board and each institution, were posted in the outpatient clinic and online community. Interested participants were provided with a study consent form and an explanatory document, and this study was initiated after this study’s details were explained to them. Since the interviews or surveys of this study were conducted online, an exemption from written consent was obtained.

In steps 2-1 and 2-3, respectively, 6 patients with IBD were recruited [25] to conduct FGIs to evaluate the qualitative appropriateness of the draft version of the scale. Participants in the FGIs were compensated with approximately US $77 (₩100,000) each. In step 3-1, the target sample size had been determined as 440, based on a calculation of 10 times the number of items in the fourth draft version of the scale, while accounting for an anticipated dropout rate of 10% [26].

Experts were recruited in steps 2-1 and 2-2 through snowball sampling to evaluate FGI and content validity. A total of 6 experts were recruited in step 2-1, including 5 nursing informatics experts and 1 medical informatics expert. Experts who participated in content validity verification received approximately US $154 (₩200,000) each. In step 2-2, eight experts were recruited, including 4 nursing informatics experts, 1 medical informatics expert, 1 educational expert, 1 gastroenterology physician, and 1 user experience designer. Patients who participated in the online survey in step 3-1 were compensated with the equivalent of approximately US $23 (₩30,000).

### Development of a Digital Health Readiness Scale

**Figure 1:**
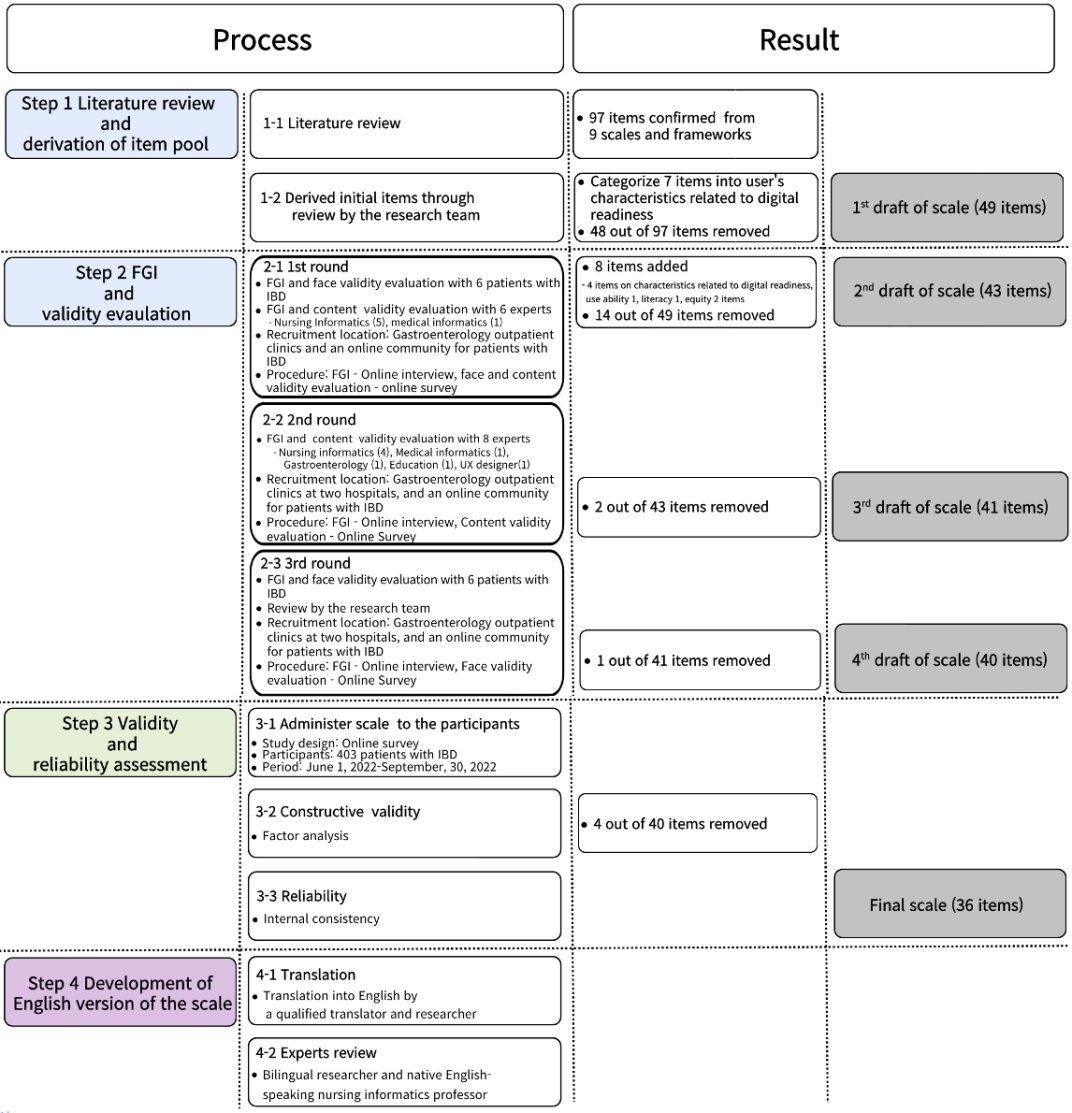
Study process and results of each step: development and validation of the Mobile-Centered Digital Health Readiness Scale for patients with inflammatory bowel disease. The names of the items reflect those of the initial version of the tool, which may differ from those of the final tool. FGI: focus group interview; IBD: inflammatory bowel disease; UX: user experience.

The primary objective of this study was to develop and validate a comprehensive scale for evaluating an individual’s readiness to engage with digital health services. The instrument focuses on assessing digital health literacy and equity among users. The overall process of scale development and the results of each step are shown in Figure 1.

#### Step 1: Literature Review and Derivation of an Initial Item Pool

##### Step 1-1: Literature Review

In our literature review process, we conducted a comprehensive search using both English and Korean languages, leveraging databases such as the Web of Science and the Korean database Research Information Sharing Service. Our search strategy focused on identifying literature and frameworks related to digital health literacy, digital health equity, and other relevant terms to ensure inclusivity and comprehensiveness, particularly concerning mHealth services. The timeframe for our literature review spanned from October 2021.

##### Step 1-2: Derived Initial Items Through Review by a Research Team

To derive items, the research team reviewed the subdomains identified in the literature review to determine the subdomains of this tool. Accordingly, the items derived in the previous stage were classified into each subdomain, and inappropriate items were excluded after reviewing their appropriateness and validity.

#### Step 2: FGI and Validity Assessment

##### Step 2-1: First Round

FGI was conducted with 6 patients with IBD to assess the face validity of the initial scale items and to gather qualitative insights regarding their relevance. These interviews were conducted online and spanned approximately 2 hours. The methodology involved structured open-ended questions, probing for understanding and appropriateness of the scale items. The process continued until data saturation was reached, indicated by the absence of new emerging data [27]. The questions were “Do you comprehend the items presented in the scale?” “In cases of lack of understanding, could you specify which aspects are unclear?” “Do you find the items within the scale to be suitable and relevant?” “If you perceive any items as unsuitable, could you elaborate on the reasons?” “Are there any elements or items that you believe should be added to enhance the scale?” and “Overall, what are your impressions or thoughts regarding the preliminary version of the scale?” In addition, FGI and content validity evaluation were conducted on 6 experts (5 in nursing informatics and 1 in medical informatics). Based on the above results, the second draft version was completed.

##### Step 2-2: Second Round

In this stage, 8 experts conducted FGI and content validity evaluation on the draft derived from the previous stage. The 8 experts comprised 4 in nursing informatics, 1 in medical informatics, 1 gastroenterology professor, 1 education doctorate, and 1 user experience designer. Then, the third draft of the scale reflecting experts’ opinions was derived.

##### Step 2-3: Third Round

In steps 2-3, FGI and face validity evaluation were conducted for 6 patients with IBD using the draft scale derived in the previous step using the same method as step 2-1. The research team reviewed the revised scale reflecting patient opinions and completed the fourth draft.

#### Step 3: Validity and Reliability Assessment

##### Step 3-1: Administer Scale to the Participants

An online survey was administered to 440 patients with IBD using the second draft of the scale derived in the earlier stage.

##### Step 3-2: Construct Validity

The construct validity was ascertained by examining the correlation between individual items and the overall scale score. A confirmatory factor analysis followed this.

##### Step 3-3: Reliability Analysis

The Cronbach α value was used to confirm reliability: if it was 0.75 or higher, it was evaluated as satisfactory, and if it was 0.6 or higher, it was evaluated as acceptable [26].

#### Step 4: Development of an English Version of the Scale

##### Step 4-1: Translation

After the reliability assessment, the final scale was concurrently developed in English and Korean to facilitate future translations. The Korean version was initially crafted, referenced by a translation into English adhering to part of World Health Organization (WHO) translation guidelines [28].

##### Step 4-2: Experts Review

Bilingual researchers specializing in medical informatics and nursing informatics reviewed the translated version of steps 4-1. Finally, a native English-speaking nursing informatics professor conducted a thorough review and revision, culminating in the finalized English scale version.

#### Statistical Analysis

The scale development involved conducting a literature review, classifying the collected content, and deriving categories through analysis of the scale developed from the literature. The scale was evaluated for face validity targeting patients with IBD, and its appropriateness was verified through expert content validity index (CVI) evaluation. The final developed scale was conducted through a survey, and the survey results were analyzed using SPSS (version 21.0; IBM Corp) as follows: first, the demographic characteristics of the participants were analyzed by frequency, percentage, average, and SD. Second, for the construct validity of the developed scale, item analysis was performed by calculating the correlation coefficient between individual items and the overall total score, and exploratory factor analysis was performed. Exploratory factor analysis was verified using principal component analysis and varimax rotation. Convergent validity was judged using the values derived through exploratory factor analysis, and the final questions were confirmed. Third, the Cronbach α was used to confirm the internal consistency of the developed scale.

#### Ethical Considerations

This study was conducted after obtaining approval (H-2108-238-1251) from the Seoul National University Hospital Bioethics Review Committee before starting this study to ensure the ethical protection of the research participants. All study participants received an explanation of this study and completed written consent. All data collected during this study period were protected through appropriate safeguards. Additionally, compensation was provided to all participants.

## Results

### Step 1: Literature Review and Derivation of an Initial Item Pool

#### Step 1-1: Literature Review

A thorough literature review was conducted to identify existing scales relevant to developing a digital health readiness evaluation scale for patients. The focus was primarily on mHealth literacy and digital health equity scales. A total of 6 scales and 3 frameworks on digital health readiness deemed suitable for this study were selected [8,29-36], and 97 items were confirmed from these scales and frameworks.

#### Step 1-2: Derived Initial Items Through Review by a Research Team

At this stage, we reviewed the subdomains derived from the literature review and determined 4 subdomains: familiarity, importance, equity, and usability (literacy). After classifying the 97 items derived from the previous stage into these 4 subdomains, we reviewed the appropriateness and validity of the items, excluding inappropriate ones. Additionally, we classified 7 user characteristics identified as important in the digital readiness framework into one additional subdomain and decided to include them in the final scale.

### Step 2: FGI and Validity Assessment

#### Step 2-1: First Round

Table 1 presents the results of face validity evaluation and FGI performed on 6 patients with IBD. This process was instrumental in identifying items irrelevant to digital health readiness or requiring further clarification. The draft scale’s CVI was rigorously assessed by 6 experts, with a threshold of 0.8 indicating high validity [37]. In addition, FGI was used to identify essential domains of digital health readiness not included in this scale and to evaluate whether the readability and language used were appropriate for the participants or data collectors [38]. As a result of the expert CVI evaluation, 20 out of 78 items were confirmed to be less than 0.8. These items were partially revised and supplemented based on expert opinions. Comments on modifying items identified through FGI included providing commonly used terms and examples to aid respondents’ understanding. Moreover, duplicate items and items that did not fit the current medical environment were deleted. As a result, 14 of the existing 49 items were deleted, and 8 items (1 item on usability, 1 item on literacy, 2 items on equity, and 4 items on characteristics related to digital readiness) were added, completing the second draft with a total of 43 items. It was decided to classify 4 digital-related characteristics items added at the expert’s suggestion and 1 item among the existing scale into the digital-related characteristics domain and include them in the final scale.

**Table 1:** Analysis of focus group interview and face validity for initial scale items: evaluations by patients with inflammatory bowel disease and experts on the Mobile-Centered Digital Health Readiness: Health Literacy and Equity Scalea.

Number	Item description	Participant feedback
2	You can use mobile devices to process administrative tasks and use electronic civil service services through public institution websites.	Item relevance: Questioned the significance of differentiating equity based on the usage of electronic civil services.
12	You can contact health-related people and send files through Social Network Services such as mobile KakaoTalk and Facebook (Meta).	Item relevance: It was noted that while proficient mobile users generally use apps effectively, there may be exceptions where skilled mobile users do not engage with social networking services.
18	Compare sources of health information and confirm whether the information is true.	Item relevance: Raised doubts about the relevance of questions about the evaluation of comments or information sources.
34	For digital health equity, health care providers (doctors, nurses, etc) need to receive related education.	Emphasized the need for equal opportunities for those unable to use digital devices, but highlighted the challenge in assessing this through questionnaire items.
35	For digital health equity, medical consumers (patients) need to receive related education.	Emphasized the need for equal opportunities for those unable to use digital devices, but highlighted the challenge in assessing this through questionnaire items.
36	For digital health equity, those involved in medical service development (health app designers, mobile medical device developers) need to receive related education.	Emphasized the need for equal opportunities for those unable to use digital devices, but highlighted the challenge in assessing this through questionnaire items.

^a^Overall feedback: Understanding of items: Participants found the terminology in the items complex and suggested modifications for easier comprehension by a broader audience. Item relevance: Expressed uncertainty about the significance of some items concerning digital literacy and equity.

#### Step 2-2: Second Round

In the expert CVI evaluation, 2 out of 78 items were confirmed to be less than 0.8 and were deleted. There were opinions that the items related to digital health equity in FGI were ambiguous, so some items were modified. Through this stage, the third draft with a total of 41 items was completed.

#### Step 2-3: Third Round

Face validity and FGI were performed on 6 patients with IBD. We collected opinions on the third draft derived from the previous stage and modified the scale by reflecting opinions on adding additional explanations to some terms and changing them with more accessible terms. Afterward, through review by the research team, one question inappropriate for measuring digital readiness was deleted. As a result, the fourth draft of 40 items was completed.

### Step 3: Validity and Reliability Assessment

#### Step 3-1: Administer Scale to the Participants

The survey, designed to assess the validity and reliability of the developed scale, encompassed a total of 403 participants. The demographic breakdown revealed that 68.5% (n=276) of the participants were under 40 years of age. Regarding gender distribution, 55.1% (n=222) were male. Educational background indicated that a significant majority, 75.2% (n=303), possessed at least a college degree. Detailed demographic characteristics are presented in Table 2.

**Table 2:** General characteristics of this study population (n=403): demographic and clinical information of patients with IBD^a^. Digital readiness-related characteristics had a mean of 9.98 (SD 2.74).

Variables and categories		Value, n (%)
**Age group (years)^b^**		
	≤20	131 (32.5)
	21-39	145 (36)
	40-49	69 (17.1)
	50-59	40 (9.9)
	≥60	18 (4.5)
**Gender**		
	Male	222 (55.1)
	Female	181 (44.9)
**Residence area**		
	Metropolitan area	329 (81.6)
	The other	74 (18.4)
**Education**		
	≤High school	100 (24.8)
	College	254 (63)
	≥Graduate school	49 (12.2)
**Disease excluding IBD**		
	Digestive disease	266 (66)
	Cardiovascular disease	15 (3.7)
	Hypertension	16 (4)
	Diabetes	10 (2.5)
	Hyperlipidemia	16 (4)
	Musculoskeletal disorders	12 (3)
	Kidney disease	8 (2)
	Respiratory diseases	14 (3.5)
	Others	117 (29)
**Number of concurrent diseases excluding IBD^c^**		
	None	44 (10.9)
	1	281 (69.7)
	2	67 (16.6)
	3	6 (1.5)
	≥4	5 (1.2)

^a^IBD: inflammatory bowel disease.

^b^Mean 36.24 (SD 11.75).

^c^Mean 1.13 (SD 0.71).

#### Step 3-2: Constructive Validity

**Figure 2:**
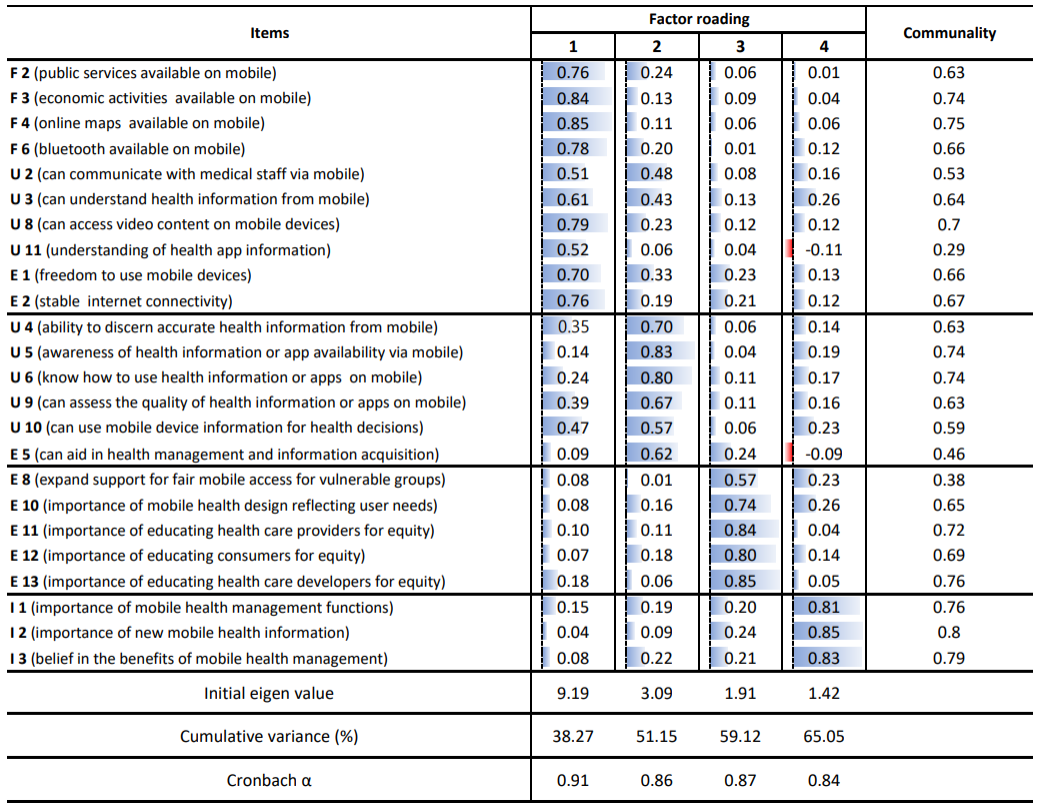
Confirmatory factor analysis and reliability assessment: evaluation of the Mobile-Centered Digital Health Readiness Scale for patients with inflammatory bowel disease.

Construct validity was verified with the fourth draft of 40 questions, and at this time, 12 questions regarding user characteristics and digital readiness-related characteristics that were decided to be included in the final questions were excluded from the analysis. Initially, the correlation coefficient between each item and the overall score was analyzed. Further, 2 items (familiarity 1 and usability 1) with correlation coefficients below 0.3 were excluded [39]. The Kaiser-Meyer-Olkin measure yielded a high value of 0.92, and the Bartlett test of sphericity was statistically significant (*χ*^2^_378_=6940.63, *P*<.001), indicating suitability for factor analysis. All factor communalities were above 0.3. In the pattern matrix, items with factor loadings above 0.5 on 2 factors and those uniquely loaded on one factor were identified. Factor analysis was iteratively performed, removing one item at a time [40]. Ultimately, 24 items loaded on 4 factors. The Kaiser-Meyer-Olkin value for the final items was 0.91, and the Bartlett test of sphericity remained significant (*χ*^2^_276_= 6114.77, *P*<.001). The commonalities of the items were above 0.38, except for one item in factor 1. These 4 factors explained 65.05% of the total variance (Figure 2). Despite low communality, one item from factor 1 was retained after consideration by the research team due to its relevance to the scale. The domain of the digital health readiness scale was named factor 1 as “capability to use mobile services,” factor 2 as “mHealth literacy,” factor 3 as “digital health equity,” and factor 4 as “perception of the importance of mHealth apps and devices” according to the content and characteristics of the loaded items.

#### Step 3-3: Reliability

The internal consistency of the final scale was evaluated using Cronbach α, as shown in Figure 2. Cronbach α for each factor ranged from 0.84 to 0.91, indicating established reliability and acceptability of the newly developed scale [26].

This scale consists of 36 items, including 24 confirmed through reliability and validity verification, and 12 items (user characteristics, characteristics related to digital readiness) that were retained in the final scale during the scale development process (Textbox 1 and Multimedia Appendix 1). Excluding the 12 items measuring user characteristics and characteristics related to digital readiness, the remaining 24 items were structured on a 5-point Likert scale. Responses range from 1 (“not at all”) to 5 (“very much”). Additionally, 5 items dedicated to evaluating digital health readiness focus on aspects of digital health accessibility. A total of 3 items, presented as multiple-choice questions, inquire about health management, information acquisition methods, and familiarity with mobile devices used for digital health services. Further, 2 items probe the willingness to pay for mHealth care services or purchase an mHealth care device. Moreover, 7 items gather data on user demographics, including age, gender, residence, occupation, education, subjective health status, and diagnosed diseases. The scale encompasses 6 domains. The total score is computed as the mean of the scores across 4 domains, excluding the user’s characteristics and characteristics related to digital readiness. Scores range from 1 to 5, with higher scores indicating greater readiness for digital health use.

**Domains and summary of the Mobile-Centered Digital Health Readiness: Health Literacy and Equity Scale.**

**Mobile services capability (10 items)**

- This domain evaluates the respondent’s knowledge and proficiency in mobile device use and the extent of their integration into daily life.

**Mobile health (mHealth) literacy understand and use mHealth apps and devices (6 items)**

- This section assesses the respondent’s ability to comprehend and use information acquired through mobile health apps and devices.

**Perception of the importance of mHealth apps and devices (3 items)**

- This domain gauges the respondent’s perceived significance of mobile health apps and devices in health care management.

**Digital health equity (5 items)**

- This domain focuses on the environmental and resource factors influencing digital health accessibility and competency. It evaluates the respondent’s access to and capability with digital health care resources.

**Characteristics related to digital readiness (5 items)**

- This domain identifies critical characteristics associated with the respondent’s digital readiness. It includes methods of acquiring health information, familiarity with mobile devices for health information, experience in using digital health care services, and willingness to invest in and pay for digital health services. This domain assesses the respondent’s accessibility to digital health resources.

**User’s characteristics (7 items)**

- This section captures the respondent’s general demographic and socioeconomic characteristics pertinent to digital health readiness. It covered age, gender, area of residence, occupation, education, subjective health status, and diagnosed diseases.

### Step 4: Development of an English Version of the Scale

We developed an English version of our questionnaire to widen this study’s reach and accessibility. As a global language, English allows us to gather data on Digital Health Readiness from diverse populations, enhancing the applicability of our findings. Offering the questionnaire in Korean and English enables researchers worldwide to use it effectively, strengthening the global understanding of digital health readiness. This decision ensures our research’s comprehensive and international relevance, aligning with our aim to adapt the tool for broader use beyond patients with IBD.

#### Step 4-1: Translation

The final scale completed in the previous step was translated into English by a qualified translator and researcher who majored in English and nursing. After being translated independently, any differences in translation were agreed upon through online communication.

#### Step 4-2: Experts Review

Bilingual researchers in English and Korean reviewed the translation of steps 4-1. In the process, we ensured that the translation applied to English speakers and corrected any expressions or cultural differences that might convey a different meaning. The English version of the scale was finally completed after a thorough review and revision by a nursing informatics professor in the United States whose native language is English (Multimedia Appendix 2).

## Discussion

### Principal Findings

In the rapidly evolving field of digital health care, accessibility to digital health services is an essential determinant of health outcomes [41]. Consequently, the concept of digital inclusion, especially for vulnerable groups, is gaining prominence in the digital health care landscape, necessitating a thorough assessment and integration of these considerations into the design and delivery of digital health services [6]. The development of the *Mobile-Centered Digital Health Readiness: Health Literacy and Equity Scale* (mDiHERS) for patients is a pioneering effort to quantify the readiness and capability of patients to engage with digital health services. This scale is particularly relevant given the chronic nature of IBD, which necessitates ongoing and continuous management and the potential for digital tools to enhance patient autonomy and care significantly. The mDiHERS addresses a critical gap in digital health literature by providing a validated tool that can assess patients’ digital access, literacy, and equity, which are essential for the effective use of digital health services.

In the mDiHERS, some items assess users’ ability to navigate and interpret digital interfaces. This ability is increasingly essential to affording opportunities to increase reach and engagement in the digital health care service [42]. Including items requiring users to interact with actual digital device screens—such as smartphones and wearable devices—addresses a vital component of digital health literacy: the technical proficiency necessary for engaging with health apps and platforms. This approach means that the mDiHERS not only captures the subjective confidence of users in their digital capabilities but also provides an objective measure of their practical skills. In other words, the mDiHERS acknowledges this by ensuring its assessment criteria encompass users’ perceived and actual abilities to manage their health digitally. This supports prior research that objective tests may be better suited to assessing an individual’s skills [10]. This is particularly pertinent for patients who rely on digital health monitoring and management tools, as it empowers them to become active, informed participants in their health care journeys. By emphasizing both confidence and competence, the mDiHERS aligns with the goals of digital health initiatives which aim to enhance patient autonomy and improve health outcomes through technology. This focus reflects the broader objectives within the digital health ecosystem, where patient empowerment and the democratization of health information are paramount.

The findings of this study underscore the importance of considering patient-specific factors when assessing digital readiness. The mDiHERS evaluates an individual’s ability and familiarity with using digital health services and their level of digital readiness, including the concept of equity, which has recently become necessary with the emergence of digital health services [7]. The tailored approach of the mDiHERS, focusing on patients with IBD, allows for a nuanced understanding of the challenges and opportunities within this group. It is evident that while digital health services offer immense potential, their benefits are not uniformly accessible. The mDiHERS can thus serve as a diagnostic tool to identify areas where interventions are needed to improve digital health engagement and patient outcomes. If digital health services regularly monitor digital readiness, they can easily identify users who need additional support. Furthermore, it can also effectively evaluate interventions’ effectiveness at all research stages. Therefore, the needs of digital health service users will be appropriately addressed.

Furthermore, assessing digital device usage skills should focus on more than just the technical aspects. As items in sections E and F of the mDiHERS exemplify, factors such as a patient’s cultural and educational background can influence their ability to use digital devices, necessitating a comprehensive approach incorporating these variables. Educational support and interventions tailored to patients from cultural and educational environments with potentially lower digital device usage skills will significantly enhance digital health literacy, accessibility, and, ultimately, digital health equity [43]. The mDiHERS underscores the need for an assessment that evaluates technical skills while also considering the patient’s overall background and circumstances. This approach contributes to developing more inclusive and customized support strategies for effectively using digital health services, ensuring all patients can benefit from advancements in digital health care.

The mDiHERS has the potential to be adapted for use in other patient populations and health conditions. Its application could lead to more personalized health care, where digital tools are used to their full potential to support health consumer’s care. However, the scale also highlights the need for health care systems to address the digital divide and ensure that all patients, particularly those with chronic conditions, have the necessary skills and resources to benefit from digital health innovations. The evolution of digital health services will likely present new challenges and opportunities, making the continuous refinement and application of tools such as the mDiHERS essential for achieving equitable and effective health care delivery.

### Limitations and Recommendation

This study used online surveys and interviews. Participants with low digital literacy may have found participating difficult, possibly excluding them from the survey. Consequently, individuals with higher digital literacy might be overrepresented, leading to self-selection bias. This can hinder generalizing the findings to the entire population and can affect study results. To minimize this bias in future research, incorporating online educational interventions and paper-based surveys can help include those with low digital literacy. Promoting the study on various platforms can also improve accessibility. Recruiting a balanced number of participants across different age groups can enhance accuracy. IBD primarily affects individuals in their teens to thirties, making it challenging to have evenly distributed participants. Future research should aim to recruit a similar number of participants across different age groups to validate the tool thoroughly and improve the representativeness of the results.

Further, it is important to note that while we aimed to gather a broad range of literature and frameworks, our approach did not use a systematic review method, which may be considered a limitation. We acknowledge this limitation in our paper as it affects the comprehensiveness of our findings. Furthermore, due to the resource constraints of our study, it was only feasible to adhere partially to the WHO translation guidelines for tools. However, we suggest that future translations of the tool into other languages should follow the WHO’s translation guidelines for tools. Lastly, future studies are proposed to measure patient or health consumer digital readiness using the mDiHERS developed in this study to advance this research field. Subsequently, they should design and evaluate digital health services considering these results. This would facilitate the validation of the correlation between outcomes derived from the tool and the actual usage and effectiveness of digital health services.

### Conclusions

The mDiHERS developed for this study measures patients’ readiness and ability to use digital health services. It is particularly useful for individuals and groups requiring continuous health management, such as IBD. mDiHERS assesses digital accessibility, literacy, and equity factors, contributing to the effective use of digital health services to enhance accessibility. The development and validation of the mDiHERS highlight the importance of patients’ confidence and competence in managing their health digitally. Continuous improvements are necessary to ensure that all patients can benefit from digital health care.

## Appendix

Multimedia Appendix 1 Mobile-Centered Digital Health Readiness: Digital Health Literacy and Equity Scale (mDiHERS) Korean version.

Multimedia Appendix 2 Mobile-Centered Digital Health Readiness: Digital Health Literacy and Equity Scale (mDiHERS) English version.

## Abbreviations

CVI: content validity index
FGI: focus group interview
IBD: inflammatory bowel disease
mDiHERS: Mobile-Centered Digital Health Readiness: Health Literacy and Equity Scale
mHealth: mobile health
WHO: World Health Organization

## Data Availability

Data from this study are available upon reasonable request from the corresponding author.
