# Supplementary material for: Development and Validation of a Mobile-Centered Digital Health Readiness Scale (mDiHERS): Health Literacy and Equity Scale
Source: J Med Internet Res. 2024 Aug 13;26:e58497. doi: 10.2196/58497 (PMC11350292; doi:10.2196/58497)
Supplement: Multimedia Appendix 1 [file jmir_v26i1e58497_app1.pdf]

모바일 디지털 헬스 준비도: 디지털 헬스 문해력과 형평성 측정도구

모바일 디지털 헬스 준비도 평가 척도는 4 가지 차원으로 구분됩니다. 모든 항목은 "1 점" (전혀 아니다)에서 "5 점" (매우 그렇다) 까지 5 점 척도로 평가됩니다. 각 문항을 읽으시고 가장 정확하게 나타내는 숫자에 표시해 주십시오.

SECTION A. 모바일 서비스 사용능력 (10 문항)						
문 항		전혀 아니다	아니다	보통 이다	그렇다	매우 그렇다
1	모바일 기기로 **행정 업무 및 공공기관 전자민원 서비스를** 이용할 수 있다.	①	②	③	④	⑤
2	모바일 기기로 **경제생활 활동을 위한 온라인 뱅킹 및 인터넷 쇼핑** 등을 할 수 있다.	①	②	③	④	⑤
3	모바일 기기로 **네비게이션, 온라인 지도 서비스 (구글 맵, 다음 거리뷰, 네이버 지도 등)**를 이용할 수 있다.	①	②	③	④	⑤
4	건강 앱 사용 시 필요한 **블루투스, 파일 공유, 기록 등의 기능**을 사용할 수 있다.	①	②	③	④	⑤
5	**의료진과의 의사소통**을 위해 모바일 기기를 활용할 수 있다. (예: 주치의/간호사와의 이메일, 카카오톡, 네이버 밴드, 화상 통화)	①	②	③	④	⑤
6	모바일 기기 및 앱을 통하여 획득한 **건강 정보를 이해**할 수 있다.	①	②	③	④	⑤
7	모바일 기기로 **건강, 운동, 교육 등을 위한 영상 컨텐츠 서비스(YouTube 등)**을 이용할 수 있다.	①	②	③	④	⑤
8	원하면 언제든지 모바일 기기를 통해 **건강관리 및 건강 정보 획득**을 위하여 **안정적인 인터넷 통신 (Wi-Fi, 3G/4G/5G/LTE)**에 접속할 수 있다.	①	②	③	④	⑤
9	원하면 **언제 어디서나 건강관리 및 건강 정보 획득**을 위하여 모바일 기기를 사용할 수 있다.	①	②	③	④	⑤

10. [모바일 헬스케어 서비스]

건강관리 앱의 화면을 보고 활용되는 기능에 대하여 어떤 의미를 가지고 있는지 알 수 있는가? 아래 4 개의 화면을 보고 활용되는 기능의 의미를 파악할 수 있다. (　　　　점/ 5 점)

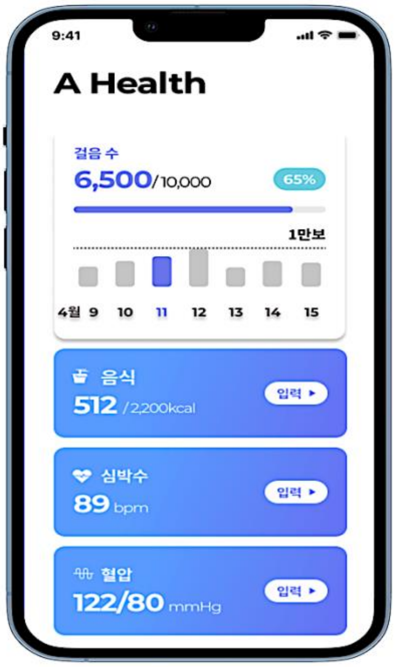

10-1. 화면에서 **심박수**를 적으세요.
(**숫자**만 작성, 모르면 **'모름'**으로 작성)

답변: ____________

10-2. 걸음수가 1 만보에 도달한 날짜를 적으세요.
(예: **0 월 0 일**, 모르면 **'모름'**으로 작성)

답변: ____________

WITH LAB
Warm IT for Healthcare Laboratory

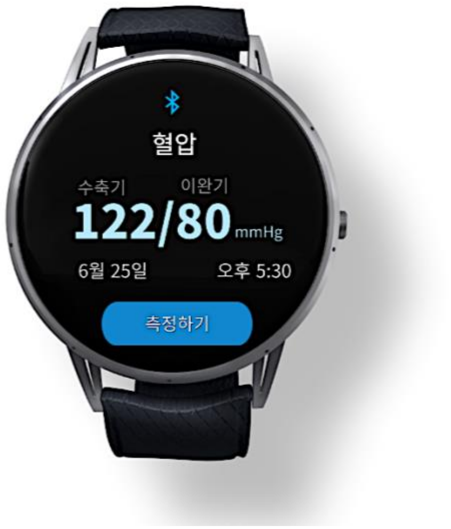

10-3. 이 화면의 정보 중 <u>**틀린**</u> 내용을 선택하세요.

① 이 화면은 **웨어러블** 기기 (예: 애플워치, 갤럭시워치, 미밴드 등)의 화면이다.
② 혈압을 측정하려면 **'측정하기'** 버튼을 누른다
③ 이 기기는 **블루투스**로 연결되어 있다.
④ 이완기 혈압은 **122**mmHg 이다.
⑤ 모르겠다.

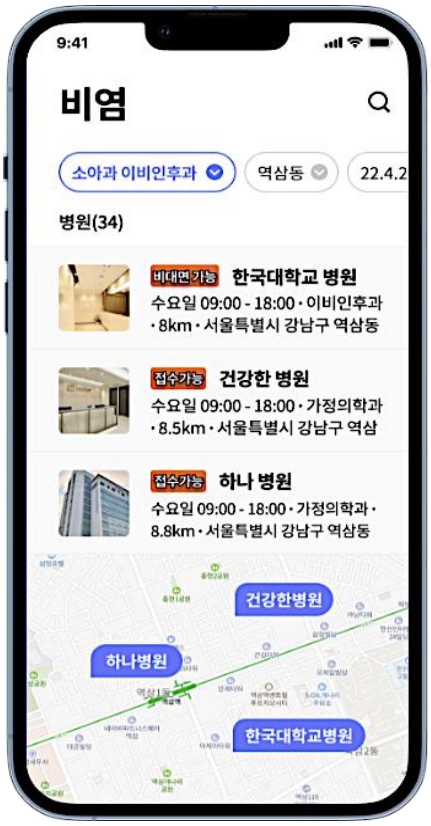

10-4. 다음 중 <u>**틀린**</u> 내용을 선택하세요.

① 이 앱은 **병원진료를 예약**하는 앱이다.
② **하나 병원**은 **방배동**에 있다.
③ 이 **화면은 비염을 진료하는 병원**을 검색한 결과이다.
④ **한국대학교 병원**은 **비대면진료**가 가능하다.
⑤ 모르겠다.

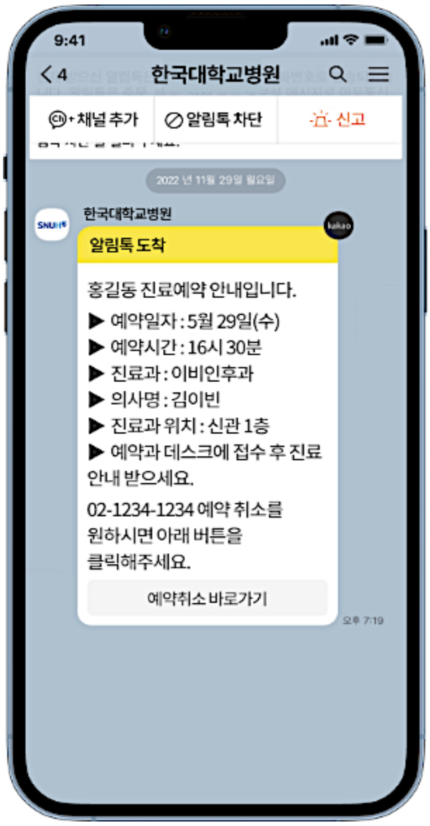

10-5. 홍길동님의 **예약시간**을 적어주세요.
(예: **0 시 0 분**. 모르면 '**모름**'으로 작성)

답변: ____________________

A. 모바일 서비스 사용능력 평균 값 =

SECTION B. 모바일 헬스 문해력: 모바일 건강 앱과 기기 이해와 활용 (6 문항)						
문 항		전혀 아니다	아니다	보통 이다	그렇다	매우 그렇다
1	모바일 앱과 기기를 통해 얻은 여러 건강 정보를 비교하여 **올바른 정보를 판별**할 수 있다.	①	②	③	④	⑤
2	모바일 기기를 통해 **어떠한 건강정보/앱 이용이 가능**한지 알고 있다.	①	②	③	④	⑤
3	모바일 기기로 찾은 **건강 정보/앱의 활용법**을 알고 있다.	①	②	③	④	⑤
4	모바일 기기를 통해 찾은 **건강 정보/앱의 질을 평가**할 수 있다.	①	②	③	④	⑤
5	**건강 관련 의사결정**을 위해 모바일 기기를 통해 얻은 **건강 정보를 활용**할 수 있다.	①	②	③	④	⑤
6	원하면 언제 어디서나 **건강관리 및 건강 정보 획득에 도움을 줄 수 있는 사람 또는 기관**이 있다(예: 건강 앱에 대한 설명이 가능한 가족 또는 보건소 등).	①	②	③	④	⑤
**B. 모바일 헬스 문해력 평균 값 =**						

SECTION C. 모바일 헬스 앱 및 기기의 중요성 인식 (3 문항)						
문 항		전혀 아니다	아니다	보통 이다	그렇다	매우 그렇다
1	모바일 기기 및 앱을 통한 **건강관리기능**은 **중요**하다.	①	②	③	④	⑤
2	건강관련 모바일 기기 및 앱에 대한 **새로운 정보**는 **중요**하다.	①	②	③	④	⑤
3	건강관리 기기 및 앱이 **나에게 도움**을 줄 수 있다고 **믿는다**.	①	②	③	④	⑤
**C. 모바일 헬스 앱 및 기기의 중요성 인식 평균 값 =**						

SECTION D. 디지털 헬스 형평성 (5 문항)						
문 항		전혀 아니다	아니다	보통 이다	그렇다	매우 그렇다
**1**	모바일 기기를 통한 건강관리 및 건강 정보에 접근하기 어려운 **취약계층에게 사회적/경제적 지원**을 **확대**하여 **공평한 접근 기회**를 **제공**해야 한다.	①	②	③	④	⑤
**2**	**사용자의 요구도를 반영**하기 위하여, **의료진과 환자가 함께** 모바일 건강관리 프로그램 및 건강데이터 관리를 **계획**하는 것은 중요하다.	①	②	③	④	⑤
**3**	디지털 헬스 형평성을 위하여, **건강관리 제공자 (의사, 간호사 등)**가 **관련 교육**을 받는 것은 중요하다.	①	②	③	④	⑤
**4**	디지털 헬스 형평성을 위하여, **의료소비자(환자)**가 **관련 교육**을 받는 것은 중요하다.	①	②	③	④	⑤
**5**	디지털 헬스 형평성을 위하여, **의료서비스 개발 관련 종사자(건강 앱 디자이너, 모바일 의료기기 개발자)**가 **관련 교육**을 받는 것은 중요하다.	①	②	③	④	⑤
**D. 디지털 헬스 형평성 평균 값 =**						

점수계산

A: 모바일 서비스 사용능력 평균 =

B: 모바일 헬스 문해력 평균 =

C: 모바일 헬스 앱 및 기기의 중요성 인식 평균 =

D: 디지털 헬스 형평성 평균 =

모바일 디지털 헬스 준비도 점수: (A+B+C+D) 점수의 평균 = (__________ 점 / 5 점)

WITH LAB
Warm IT for Healthcare Laboratory

SECTION E. 디지털 준비도 관련 특성 (5 문항)

1) 건강관리 및 건강정보를 습득하기 위해 **주로** 어떤 **방법**을 사용하는가? (복수 선택 가능)

① 디지털 기기 (스마트폰, 테블릿 PC, 노트북 등)
② 방송매체 (TV, 라디오 등)
③ 인쇄매체 (신문, 책 등)
④ 헬스 케어 전문가 (의사, 간호사 등)
⑤ 사회적 인간관계 (가족, 지인 등)
⑥ 기타 ( )

2) 건강관리 및 건강정보 습득을 위하여 비교적 **친숙한 모바일 기기**는 무엇인가? (복수 선택 가능)

① 스마트폰
② 태블릿 PC (아이패드, 갤럭시 탭 등)
③ 컴퓨터/노트북
④ 웨어러블 기기 (스마트 밴드, 갤럭시 워치 등)
⑤ 모바일 의료기기 (블루투스 혈압계/혈당계 등)
⑥ 기타 ( )

3) 건강관리 및 건강정보 습득을 위하여 사용해본 **경험**이 있는 **디지털 건강관리 서비스**는 무엇인가? (복수 선택 가능)

① 스마트폰에 설치되어 있는 건강 앱 (삼성헬스 앱, 애플 건강 앱 등)
② 직접 설치/다운로드한 건강 앱 (다이어트 앱, 당뇨관리 앱 등)
③ 온라인 강의/건강관리 프로그램 (환자 식단관리 강의, 약물정보 강의, 온라인 환우회 등)
④ 1:1 원격진료/화상상담
⑤ SNS (카카오톡, 네이버밴드, 인스타그램 등)
⑥ 기타 ( )
⑦ 경험 없음

4) 모바일 기기를 통한 건강관리 및 건강정보 제공_**서비스**에 **비용**을 **지불**할 의사가 있는가?
(예: 비만 앱과 걸음 수 추적이 가능한 스마트 워치를 활용한 체중 관리 4 주 프로그램, 염증성 장 질환관련 개인화된 정보제공 앱 기반 4 주 증상 기록 프로그램)

① 비용지불 의사 없음
② 1 천원/월
③ 5 천원/월
④ 1 만원/월
⑤ 1 만원/월 초과

5) 건강관리를 위한 모바일 **기기 구입에 비용**을 지불할 의사가 있는가?
(예: 심박수, 걸음 수, 수면 기록, 심전도 및 산소포화도 등이 측정 가능한 스마트 워치)

① 비용지불 의사 없음
② 1~5 만원
③ 6~10 만원
④ 11~15 만원
⑤ 16 만원 이상

.

WITH LAB
Warm IT for Healthcare Laboratory

SECTION F. 사용자 특성 (7 문항)

1) 본인의 **생년월일**을 적어주세요. (예: 1986-03-14)

(__________________)

2) 본인의 **성별**을 선택하세요.

① 남성 ② 여성 ③ 기타 ( ) ④ 밝히고 싶지 않습니다.

3) 현재 **거주지역**을 선택하세요.

① 서울특별시 ② 경기도 ③ 인천광역시 ④ 강원특별자치도
⑤ 충청도 ⑥ 경상도 ⑦ 전라도 ⑧ 제주특별자치도

4) 본인의 **직업**을 선택하세요.

① 무직 ② 전문가 및 관련 종사자 ③ 사무 종사자
④ 서비스 종사자 ⑤ 판매 종사자 ⑥ 농림어업 숙련 종사자
⑦ 기능원 및 관련기능 종사자 ⑧ 장치, 기계 조작 및 조립 종사자 ⑨ 단순노무 종사자
⑩ 군인 ⑪ 관리자 ⑫ 기타 ( )

5) 본인의 **최종학력**을 선택하세요.

① 초등학교 졸업 ② 중학교 졸업 ③ 고등학교 졸업
④ 대학교 졸업 ⑤ 대학원 이상 ⑥ 기타 ( )

6) 귀하가 판단하는 **현재 전반적인 건강상태**는 몇 점인가요?
(가장 좋지 않은 건강상태 '0'이고 가장 좋은 건강상태는 '10' 입니다)

0 ----- 1 ----- 2 ----- 3 ----- 4 ----- 5 ----- 6 ------ 7 ----- 8 ----- 9 ----- 10

7) **현재까지 진단받은 질환**을 모두 고르세요 (복수 선택 가능).

① 고혈압 ② 당뇨 ③ 심혈관질환 (협심증, 심근경색 등)
④ 뇌혈관질환 (뇌경색, 뇌출혈 등) ⑤ 암 ⑥ 기타 ( )

- 생년월일, 거주지역 등의 문항은 연구 상황, 국가에 따라 변경해서 사용가능
