# Supplementary material for: Development and Validation of a Mobile-Centered Digital Health Readiness Scale (mDiHERS): Health Literacy and Equity Scale
Source: J Med Internet Res. 2024 Aug 13;26:e58497. doi: 10.2196/58497 (PMC11350292; doi:10.2196/58497)
Supplement: Multimedia Appendix 2 [file jmir_v26i1e58497_app2.pdf]

Mobile-centered Digital Health Readiness: Digital Health Literacy and Equity Scale (mDiHERS)

The Mobile-centered Digital Health Readiness Assessment Scale is divided into four dimensions.
All items are rated on a 5-point scale from “1” (not at all) to “5” (very much). Please read each item and indicate a number.

SECTION A. Mobile Services Capability [10 Items]						
Question		Strongly Disagree	Disagree	Neutral	Agree	Strongly Agree
1	I can access **government services and electronic civil services of public institutions** on mobile devices.	①	②	③	④	⑤
2	I can use mobile devices for **economic activities such as mobile banking and online shopping.**	①	②	③	④	⑤
3	I can **use online maps and navigation services (Google Maps, Apple Maps, etc.)** on mobile devices.	①	②	③	④	⑤
4	I can use **Bluetooth, file sharing, and recording functions** when using the health app.	①	②	③	④	⑤
5	I can use mobile devices to **communicate with medical staff** (Example: email, KakaoTalk, Naver Band, video call with doctor/nurse).	①	②	③	④	⑤
6	I can **understand health information** obtained through mobile apps and devices.	①	②	③	④	⑤
7	I can use video **content services (such as YouTube) for health, exercise, and education** on my mobile device.	①	②	③	④	⑤
8	I can **access stable Internet communication (Wi-Fi, 3G/4G/5G/LTE)** on mobile devices **for my health management and health information acquisition.**	①	②	③	④	⑤
9	I can use mobile devices **to manage my health and obtain health information anytime, anywhere.**	①	②	③	④	⑤

10. [Mobile Healthcare Services]

Can you determine the meaning of each function by looking at the screen of the health care app?
You can understand the meaning of the functions being used by looking at the four screens below.

(score/ 5 score**)**

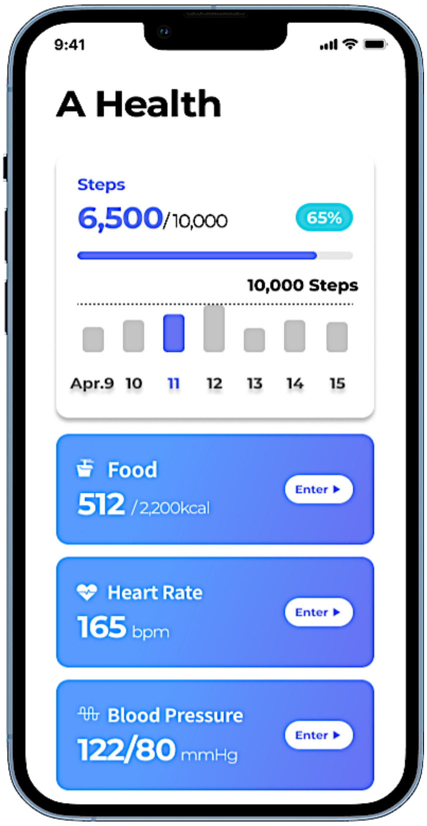

10-1. Please enter the **"heart rate"** shown on the screen.
(Write a **number** only; **"don't know"** if you don't know).

Answer: ______________

10-2. Please enter the date when you reached 10,000 steps.
(e.g., **MM/DD**; write **"don't know"** if you don't know)

Answer: ________________

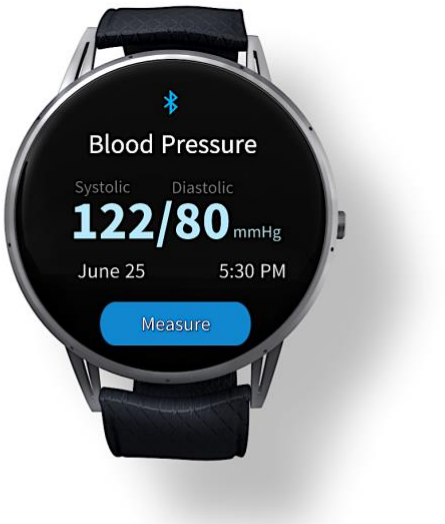

10-3. Please select the **incorrect** information on this screen.

① This screen is the screen of a **wearable device** (e.g. Apple Watch, Galaxy Watch, Mi-Band, etc.).
② Press **"Measure"** to measure blood pressure.
③ This device is connected via **Bluetooth.**
④ The diastolic blood pressure is **122** mmHg.
⑤ I don't' know.

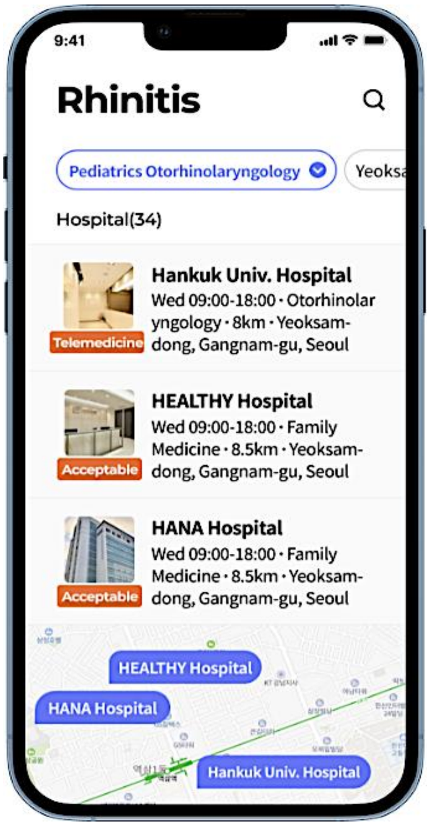

10-4. Please choose the **incorrect** information.

① The app allows you to **schedule hospital appointments.**
② **HANA Hospital** is located in **Bangbae-dong.**
③ This screen is the search results **for hospitals that treat rhinitis.**
④ **Hankuk University Hospital** is available for **non-face-to-face consultation (telemedicine).**
⑤ I don't' know.

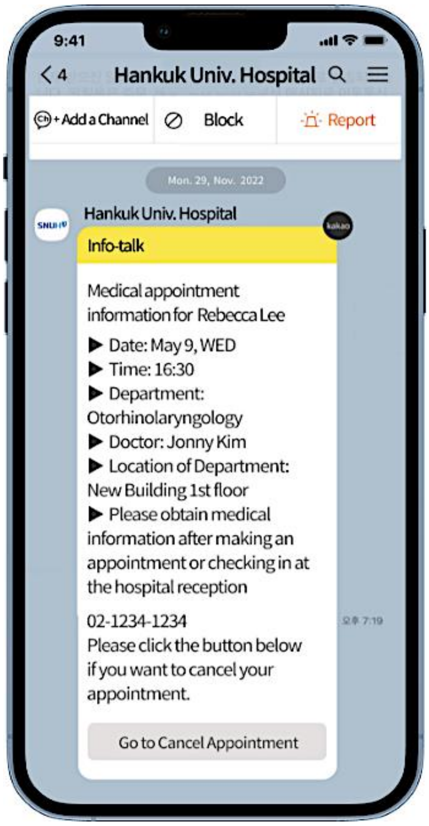

10-5. Please enter the **reservation time** for Rebecca Lee.
(e.g., **00:00**; write **"don't know"** if you don't know)

Answer: ____________________

A. Mobile services capability mean score =

SECTION B. mHealth Literacy: Understand and utilize mobile health apps and devices [6 Items]						
Question		Strongly Disagree	Disagree	Neutral	Agree	Strongly Agree
1	You can **compare various health information obtained through mobile devices and apps** to determine accurate information.	①	②	③	④	⑤
2	I am **aware of what health information** and app usage are possible through mobile devices.	①	②	③	④	⑤
3	I am **familiar with the utilization** of health information and apps found through mobile devices.	①	②	③	④	⑤
4	You can **assess the quality of health information/apps** found through mobile devices.	①	②	③	④	⑤
5	You can **utilize health information** obtained through mobile devices for **health-related decision-making.**	①	②	③	④	⑤
6	There are **individuals or institutions available anytime, anywhere, if desired, to assist with health management and obtaining health information** (e.g., family members who can provide explanations about health apps or public health clinics).	①	②	③	④	⑤
**B. mHealth Literacy mean score =**						

SECTION C. Perception of the importance of mHealth apps and devices [3 Items]						
Question		Strongly Disagree	Disagree	Neutral	Agree	Strongly Agree
1	**Health management function** on mobile devices and apps are important.	①	②	③	④	⑤
2	**New information** about health-related mobile devices and apps is important.	①	②	③	④	⑤
3	I **believe** that health management devices and apps **can help me.**	①	②	③	④	⑤
**C. Perception of the importance of mHealth apps and devices mean score =**						

SECTION D. Digital Health Equity [5 Items]						
Question		Strongly Disagree	Disagree	Neutral	Agree	Strongly Agree
**1**	It is necessary to provide **equal access opportunities** by **expanding social and economic support to vulnerable people** who have difficulty accessing healthcare services and health information through mobile devices.	①	②	③	④	⑤
**2**	**To meet user needs**, it is important for **healthcare professionals and patients to collaborate in planning** mobile health management programs and health data management**.**	①	②	③	④	⑤
**3**	For digital health equity, it is important for **healthcare providers (doctors, nurses, etc.) to receive relevant education.**	①	②	③	④	⑤
**4**	For digital health equity, it is important for **healthcare consumers (patients) to receive relevant education.**	①	②	③	④	⑤
**5**	For digital health equity, it is important for **healthcare service development professionals (health app designers, mobile medical device developers, etc.) to receive relevant education.**	①	②	③	④	⑤
**D. Digital Health Equity mean score =**						

Scoring

A: Mobile Services Capability mean score =

B: mHealth Literacy mean score =

C: Perception of the importance mean score =

D: Digital Health Equity mean score =

Mobile Digital Health Readiness (A+B+C+D) mean score = (score / 5 score)

WITH LAB
Warm IT for Healthcare Laboratory

SECTION E. Characteristics related Digital Readiness [5 Items]

1) What **methods** do you **mainly** use to manage your health management and health information? (Multiple choice allowed)

① Digital devices (smart phones, tablet PC, laptops, etc.)
② Broadcasting media (TV, radio, etc.)
③ Print media (newspapers, books, etc.)
④ Healthcare professionals (doctors, books, etc.)
⑤ Social relationships (family, acquaintances, etc.)
⑥ Other (   )

2) What are relatively **familiar mobile devices** for health management and acquiring health information? (Multiple choice allowed)

① Smart phone
② Tablet PC (iPad, Galaxy Tab, etc.)
③ Computer/laptop
④ Wearable device (Galaxy Watch, smart band, etc.)
⑤ Mobile medical devices (bluetooth blood pressure monitor/glucose meter, etc.)
⑥ Other (   )

3) What **digital health management services** have you tried for health management and **acquiring** health information? (Multiple choice allowed)

① Health apps installed on your smartphone (Samsung Health, Apple Health app, etc.)
② Self-installed/downloaded health apps (diet apps, diabetes management apps, etc.)
③ Online lectures/health management programs
(patient diet management lectures, drug information lectures, online patient associations, etc.)
④ 1:1 telemedicine/video consultation
⑤ SNS (Kakao Talk, Facebook, Instagram, etc.)
⑥ Other (   )
⑦ No experience

4) Do you have a willingness to **pay** for health management and health information **services** through mobile devices? (For example, a 4-week weight management program using an obesity app and a smartwatch that tracks step count, or a 4-week symptom recording program based on a personalized information-providing app related to Inflammatory Bowel Disease.)

① No willingness to pay
② $ 1/month
③ $ 5/month
④ $ 10/month
⑤ Over $ 10/month

5) Do you have a willingness to pay for the **purchase of mobile devices** for health management? (For example: a smartwatch capable of measuring heart rate, step count, sleep patterns, electrocardiogram, and oxygen saturation)

① No willingness to pay
② $ 10 to 50
③ $ 51 to 100
④ $ 101 to 150
⑤ Over $ 151

WITH LAB
Warm IT for Healthcare Laboratory

SECTION F. User's Characteristics [7 Items]

1) Please write down your **date of birth**. (Ex: 1986-Mar-14)

(__________________)

2) Please select your **gender**.

① Male ② Female ③ Other ( ) ④ I prefer not to disclose.

3) Please select your current **place of residence**.

① Seoul ② Gyeonggi-do ③ Incheon ④ Gangwon State
⑤ Chungcheong-do ⑥ Gyeongsang-do ⑦ Jeolla-do
⑧ Jeju Special Self-Governing Province

4) Please select your **occupation**.

① Unemployed ② Professionals and related occupation ③ Office clerk
④ Service worker ⑤ Salesperson ⑥ Skilled person in agriculture, forestry, and fisheries
⑦ Technicians and related technical workers ⑧ Machine operators and assemblers
⑨ Unskilled laborer ⑩ Soldier ⑪ Manager ⑫ Other ( )

5) Please select your **highest level of education**.

① Elementary school graduation ② Middle school graduate
③ High school graduate ④ College graduate
⑤ Graduate school or more ⑥ Other ( )

6) How would you rate your **current general well-being** on a scale of 0 to 10 as you perceive it?
(On a scale where 10 represents the best possible health and 0 represents the worst possible health).

0 ----- 1 ----- 2 ----- 3 ----- 4 ----- 5 ----- 6 ------ 7 ----- 8 ----- 9 ----- 10

7) Please select **all diseases diagnosed.** (Multiple choices allowed.)

① Hypertension ② Diabetes mellitus
③ Cardiovascular diseases (angina pectoris, myocardial infarction, etc.)
④ Cerebrovascular diseases (cerebral infarction, cerebral hemorrhage, etc.)
⑤ Cancer ⑥ Other ( )

- Questions regarding date of birth, area of residence, and similar items may be modified according to the research context and the country of use.
